# Breastfeeding during the COVID-19 pandemic: what do we know so far?

**DOI:** 10.31744/einstein_journal/2022RW6609

**Published:** 2022-07-04

**Authors:** Taison Regis Penariol Natarelli, Heloisa Gasparini Marigheti Brassarola, Luciana Mara Monti Fonseca

**Affiliations:** 1 Escola de Enfermagem de Ribeirão Preto Universidade de São Paulo Ribeirão Preto SP Brazil Escola de Enfermagem de Ribeirão Preto, Universidade de São Paulo, Ribeirão Preto, SP, Brazil.

**Keywords:** Breast feeding, Lactation, Milk, human, COVID-19, Coronavirus infections, Betacoronavirus, SARS-CoV-2, Infant, newborn, Child health, Protective factors

## Abstract

**Objective:**

This study aimed to conduct a literature review on safety in breastfeeding in mothers with COVID-19. An integrative review of national and international literature was carried out.

**Methods:**

The search took place in the SciELO, LILACS and MEDLINE^®^ databases.

**Results:**

A total of 25 scientific articles that specifically addressed the theme of breastfeeding and its risk and protective factors for infection by SARS-CoV-2 were selected. According to the studies analyzed, although the presence of viral RNA had already been detected by reverse transcriptase polymerase chain reaction in breast milk samples, there are still no proven cases of vertical transmission via human milk.

**Conclusion:**

Breastfeeding appears to be safe if practiced according to precaution measures recommended by the World Health Organization. In addition, there is evidence of a potential for immunological protection by transfer of antibodies against SARS-CoV-2 in breast milk. Breastfeeding should continue to be promoted even in cases of mothers with clinical suspicion or confirmation of COVID-19, as well as the provision of expressed breast milk in cases where there is no possibility of direct breastfeeding.

## INTRODUCTION

On December 31, 2019, China informed the World Health Organization (WHO) about cases of pneumonia of unknown etiology, diagnosed in the city of Wuhan, Province of Hubei. On January 30, 2020, the WHO declared the outbreak of severe acute respiratory disease coronavirus 2 (SARS-CoV-2) as Public Health Emergency of International Concern.^(1)^ The pandemic of the coronavirus 2019 disease (COVID-19) reached the five continents, infected more than 180 million people, lead to over 4 million deaths, and has persisted until this day.

The advance of the pandemic and the spread of the new coronavirus directly impacted on the population’s living habits and on routines of health care services, including maternity services, which adopted strict precaution measures to prevent the transmission of the new and still puzzling agent. Amidst uncertainties and hypotheses, some practices, such as breastfeeding, human milk donation and skin-to-skin contact between mother and newborn, were cautiously treated by health teams, due to the possibility of transmitting SARS-CoV-2.

Breastfeeding, however, provides countless benefits to the health of children and women, in the short and long run, especially for its immunological properties, rendering it a protective factor for infant morbidity and mortality. Therefore, exclusive breastfeeding is highly recommended for the first 6 months of the child’s life and complementary feeding for up to 2 years or more.^(2)^

In suspected or confirmed cases of mothers with COVID-19, the Ministry of Health, supported by the Center for Disease Control and Prevention (CDC), recommends rooming-in for the mother-child pair, maintaining a minimum 1m, preferably 2m distance between the mother’s bed and the newborn’s crib. The Ministry of Health also provides guidelines so that, in these cases, breastfeeding is carried out with precaution measures, such as masks and hand hygiene.^(3,4)^

At the beginning of the pandemic, there were few studies regarding the possibility of transmission – vertical or horizontal – of the new coronavirus through breastfeeding. While health authorities, such as the Ministry of Health and the CDC, maintained the recommendations for promoting and guaranteeing breastfeeding during the pandemic, since the benefits of this practice outweigh any potential risks,^(3,4)^ there was consensus among experts in China to contraindicate breastfeeding in cases of infected puerperal women, based on previous experience with SARS (Severe Acute Respiratory Syndrome), and given the possibility of vertical transmission of the SARS-CoV-2.^(5)^

Given this complex scenario introduced by the COVID-19 pandemic, it is extremely necessary that the often controversial evidence regarding the possibility of vertical or horizontal transmission related to breastfeeding, as well as the presence of SARS-CoV-2 in breastmilk, be critically analyzed, discussed and disseminated to the scientific community. This effort would give support to the practice of those who have been working in maternal and child health during the pandemic, since many still have doubts about such recommendations, bearing in mind this is a new disease, with high rates of transmissibility and lethality. Its transmission, clinical consequences, and treatment are still being investigated.

## OBJECTIVE

To map scientific evidence on the possible risks of vertical or horizontal transmission of SARS-CoV-2 through breastfeeding, besides the benefits of human milk for the child’s immunologic protection against COVID-19.

## METHODS

An integrative review of national and international scientific literature was carried out, according to the following steps: theme identification and selection of the research question; determination of criteria for inclusion and exclusion of studies; classification of studies; evaluation of studies; interpretation and discussion of results; presentation of the review or synthesis of knowledge.^(6)^ For selection and inclusion of articles, the guidelines of the Preferred Reporting Items for Systematic Reviews and Meta-Analyses (PRISMA), comprising four phases, identification, selection, eligibility and inclusion were used.^(7)^

Having as a starting point the core topic of breastfeeding and COVID-19, which guided this review, the following search question was generated: What is the scientific evidence so far on the safety of breastfeeding in cases of mothers with COVID-19?. In this study, cases considered as safe were of breastfed children which had tested negative on reverse transcription polymerase chain reaction (RT-PCR) for SARS-CoV-2 or had not manifested relevant clinical signs that lead to the testing.

For the sample selection, the following inclusion criteria were established: studies that addressed the issue of breastfeeding in cases of mothers with COVID-19, its risk or protective factors, published as from 2020 to January 2021, in English or Portuguese. Literature reviews, monographs, dissertations, theses, manuals, and technical standards were excluded.

The search was carried out at the Latin American and Caribbean Literature on Health Sciences (LILACS) and MEDLINE^®^ databases, and at the Scientific Electronic Library Online (SciELO) repository. The Health Sciences Descriptors (DeCS) used were: “*aleitamento materno*” OR “breast feeding” AND “*infecções por coronavírus*” OR “coronavirus infections”.

After being selected, the articles were classified (title, journal, date of publication, country of origin and type of study), analyzed in depth, and, subsequently, the main findings were extracted, discussed, and presented as a synthesis of knowledge. The following variables were analyzed: presence or absence of SARS-CoV-2 in the breast milk; presence or absence of anti-SARS-CoV-2 antibodies in the breast milk; positive or negative RT-PCR for SARS-CoV-2 in breastfed children, and presence or absence of symptoms in breastfed children.

## RESULTS

**Figure 1:**
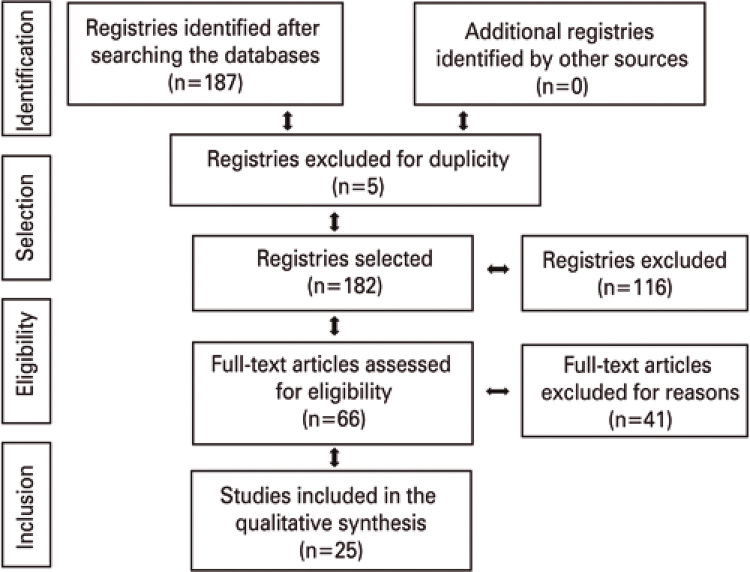
Selection of studies

A total of 187 studies were identified (7 at SciELO; 8 at LILACS, and 172 at MEDLINE^®^), of which 5 were excluded for repetition in the databases, and 116 excluded after reading the title/abstract. A total of 66 studies were pre-selected; of these, 41 were excluded after reading the full text, since they did not fit the proposed topic, totaling 25 scientific articles that were included in the review (Figure 1).

The articles selected were filed in chronological order and classified according to table 1.

**Table 1:** References included in this review

References	Country of origin	Type of study
Lang et al.^(8)^	China	Case report
Wu et al.^(9)^	China	Single center cohort study
Salvatori et al.^(10)^	Italy	Experience report
Ferrazzi et al.^(11)^	Italy	Retrospective multicenter study
Zhu et al.^(12)^	China	Retrospective study
Lowe et al.^(13)^	Australia	Case report
Pereira et al.^(14)^	Spain	Retrospective study – experience report
Marín Gabriel et al.^(15)^	Spain	Multicenter study
Yu et al.^(16)^	China	Case report
Pereira et al.^(17)^	Spain	Case series
Chambers et al.^(18)^	USA	Quantitative research (analysis of breast milk samples from mothers with COVID-19)
Marín Gabriel et al.^(19)^	Spain	Prospective observational study
Patil et al.^(20)^	USA	Retrospective cross-sectional study
Bastug et al.^(21)^	Turkey	Case report
Lebrão et al.^(22)^	Brazil	Case report
Kalamdani et al.^(23)^	India	Retrospective study – case review
Salvatore et al.^(24)^	USA	Observational cohort study
Zhao et al.^(25)^	China	Quantitative research (analysis of breast milk samples from mothers with COVID-19)
Oba et al.^(26)^	Brazil	Case report
Okeahalam et al.^(27)^	South Africa	Cross-sectional study
Perrone et al.^(28)^	Italy	Case report
Olivini et al.^(29)^	Italy	Retrospective observational study – case series
Dong et al.^(30)^	China	Case report
Lugli et al.^(31)^	Italy	Case report
Marín Gabriel et al.^(32)^	Spain	Multicenter descriptive study

Table 2 presents the key findings drawn from the studies, including the presence of viral RNA by RT-PCR testing for SARS-CoV-2 in breast milk samples from mothers with COVID-19 and the presence of anti-SARS-CoV-2 antibodies, in addition to the clinical outcome of children who were breastfed or received breast milk expressed by mothers infected with the new coronavirus.

**Table 2:** Main results of the analyzed studies

References	Human milk samples from mothers with COVID-19				Outcomes of breastfed children		
	Positive RT-PCR	Negative RT-PCR	Total n	Presence of antibodies	Positive RT-PCR	Negative RT-PCR or asymptomatic	Total n
Lang et al.^(8)^	0	1	1	-	0	1	1
Wu et al.^(9)^	1	2	3	-	0	5	5
Salvatori et al.^(10)^	0	2	2	-	2	-	2
Ferrazzi et al.^(11)^	-	-	-	-	2	8	10
Zhu et al.^(12)^	1	4	5	-	-	-	-
Lowe et al.^(13)^	-	-	-	-	0	1	1
Pereira et al.^(14)^	-	-	-	-	0	21	21
Marín Gabriel et al.^(15)^	-	-	-	-	-	19	19
Yu et al.^(16)^	0	4	4	Yes (2/2)	1	0	1
Pereira et al.^(17)^	-	-	-	-	0	20	20
Chambers et al.^(18)^	1	63	64	-	-	-	-
Marín Gabriel et al.^(19)^	0	7	7	-	-	-	-
Patil et al.^(20)^	-	-	-	-	0	31	31
Bastug et al.^(21)^	1	0	1	-	1	0	1
Lebrão et al.^(22)^	-	-	-	Yes (2/2)	0	1	1
Kalamdani et al.^(23)^	-	-	-	-	12	173	185
Salvatore et al.^(24)^	-	-	-	-	0	64	64
Zhao et al.^(25)^	0	4	4	-	-	-	-
Oba et al.^(26)^	0	2	2	-	1	0	1
Okeahalam et al.^(27)^	-	-	-	-	-	-	-
Perrone et al.^(28)^	0	1	1	-	0	1	1
Olivini et al.^(29)^	0	2	2	-	4	1	5
Dong et al.^(30)^	0	7	7	Yes (6/6)	0	1	1
Lugli et al.^(31)^	2	0	2	-	0	1	1
Marín Gabriel et al.^(32)^	-	-	-	-	0	198	198

RT-PCR: reverse transcriptase polymerase chain reaction.

Out of 105 breast milk samples from mothers with COVID-19 analyzed using the RT-PCR test, in 14 different studies, only six (5.71%) detected SARS-CoV-2 RNA.^(9,12,18,21,31)^The viral RNA was not identified in other samples (94.29%).^(8-10,12,16,18,19,25,26,28-30)^ In the study by Chambers et al.^(18)^ 64 samples of breast milk from 18 women diagnosed with COVID-19 were analyzed; however, despite having detected SARS-CoV-2 RNA in one sample, the viral culture for this same sample was negative.

Olivini et al.^(29)^ reported the case of a SARS-CoV-2 negative newborn who was isolated from the mother diagnosed with COVID-19 and fed with expressed breast milk, following precaution measures (mask and hands and breast hygiene), remaining healthy during hospital stay.

Among the studies analyzed that evaluated the risks of horizontal transmission between a mother positive and a child negative for SARS-CoV-2, in cases where breastfeeding was established in accordance to WHO and the United Nations Children’s Fund (Unicef) recommendations, newborns and infants had negative RT-PCR for SARS-CoV-2 or remained asymptomatic, or yet had mild and nonspecific symptoms, not requiring a specific test.^(8,9,11,13-15,17,20,22-24,28,30-32)^

Horizontal mother-to-child transmission has been described in case reports of newborns who were breastfed by mothers with COVID-19, not wearing masks, who subsequently tested positive for SARS-CoV-2,^(11)^ or else were kept in the same bed as the infected mothers, due to lack of physical space in rooming-in, not meeting the WHO/Unicef recommendations.^(23)^

Three studies analyzed the presence of anti-SARS-CoV-2 antibodies by means of an enzyme linked immuno sorbent assay (ELISA), identifying immunoglobulin A (IgA), immunoglobulin G (IgG) or immunoglobulin M (IgM) , in ten samples of breast milk from lactating women with COVID-19.^(16,22,30)^In a study carried out in 53 African countries, it was found that, in areas where there is early initiation of breastfeeding, there is also a lower risk of infection and death by COVID-19.^(27)^ Breastfeeding is also a protective factor in cases of co-infection by other pathogens, such as *Clostridioides difficile*.^(26)^

## DISCUSSION

The articles were mostly international (23; 92%), demonstrating a relative scarcity of national studies on the subject of breastfeeding and COVID-19. The countries with the highest number of articles selected were, in descending order: China (6; 24%), Spain (5; 20%), Italy (5; 20%), United States (3; 12%), Brazil (2; 8%), South Africa (1; 4%), Australia (1; 4%), India (1; 4%) and Turkey (1; 4%). Most of the scientific papers were retrospective studies, case/experience reports (17; 68%), with small samples. Such factors were considered limitations of the analyzed studies.

In a systematic review including 37 studies, out of a total of 84 breast milk samples from mothers diagnosed with COVID-19, nine had positive RT-PCR for SARS-CoV-2. The results of this review corroborate the findings of the present study, which also identified, for the most part, the absence of SARS-CoV-2 in human milk. However, it is important to note that viral RNA detection can be affected by milk components. Furthermore, the presence of viral RNA in the milk does not necessarily indicate viral infectivity.^(33)^

Of the five studies that identified viral RNA in breast milk, only one explained the adoption of strict precaution measures (hand and breast hygiene plus mask and gloves) to avoid contamination during sample collection.^(31)^ Recently, McGuire et al.^(34)^proposed good practices for the collection and storage of breast milk in research on COVID-19, which include the steps of breast hygiene, milk collection, portioning, and storage. The same study addressed factors that may influence the results of breast milk analyzes and should be considered by researchers, such as time from delivery (the number of antibodies varies according to the stages of lactation), and the presence of inflammation. The author also highlighted the impact of different collection techniques, since, in the case of a lipophilic virus such as SARS-CoV-2, the method of extracting milk, whether manually or using an electric pump, complete or partial expression, may also influence the identification of the pathogen, since the hind milk has a higher proportion of fat and, consequently, a greater amount of the pathogen. Disinfection of electric pumps and hand and breast hygiene are key practices to avoid sample contamination with viral RNA present on surfaces and tissues.^(34)^

The study by Pace et al.^(35)^ addressed the presence of the virus in the skin of the breast (areola and nipple), with evidence of viral RNA being detected in eight of 70 swabs, and only one positive result was considered conclusive. Based on these results, the authors do not suggest systematically washing the breasts before breastfeeding, except in cases where the mother coughs on the exposed breast, requiring cleaning with warm water and soap before breastfeeding.^(35)^

As for the presence of specific antibodies, in a study that analyzed 37 samples of breast milk from 18 mothers with COVID-19, 76% of samples contained SARS-CoV-2 specific IgA and 80% IgG, and 62% of samples were able to neutralize the infectivity of the virus *in vitro*.^(35)^ Such findings also reinforce the results of this research and the potential of breast milk as an immunological protection factor.

The results of this study coincide with the findings of other recently published reviews.^(33,36)^ Existing scientific evidence is still insufficient to prove or rule out the possibility of transmission of SARS-CoV-2 through breast milk, highlighting the need for more studies on the topic, especially large-scale cohort or case-control studies, still unpublished on this topic.^(33,36)^

Another factor that makes it difficult to determine the vertical transmission of SARS-CoV-2 through breast milk is that, when treating newborns who tested positive for COVID-19, it is difficult to state whether the source of transmission was via breast milk, respiratory droplets, transplacental or through the birth canal.^(36)^

Although it is not possible to ignore the possibility of transmission of SARS-CoV-2 through human milk, or even through skin-to-skin contact and other maternal fluids, such as blood and sweat, and given the undeniable benefits of breastfeeding , authors continue to reinforce the WHO recommendations regarding encouraging breastfeeding, whenever the health conditions of the mother and baby are adequate, and with the mother being informed on precaution measures, and about the possibility of vertical and horizontal transmission.^(33,36)^

## CONCLUSION

It is commonly assumed that breastfeeding should be encouraged even in suspected or confirmed cases of mothers with COVID-19, since no study in this review could prove cases of vertical transmission of SARS-CoV-2 through breast milk. Although the presence of viral RNA has been identified in human milk samples, apparently the virus, under these conditions, has not demonstrated the ability to replicate or infect the baby. Additionally, the presence of anti-SARS-CoV-2 antibodies in breast milk attributes to breastfeeding a protective factor against infection in children, especially against the severe form of the disease.

Both direct breastfeeding and offering expressed breast milk seem to be safe practices in cases of mothers diagnosed with COVID-19, provided that precaution measures, such as hand hygiene and mask are adopted, given that the proven benefits of breast milk still outweigh the risks of horizontal transmission. Further studies are required with stricter methods, and guided by good practices for collection and storage of breast milk in research on COVID-19, to fill gaps on safety of breastfeeding in the context of the COVID-19 pandemic.
